# Dr. Anthony Fauci Shares Insights on His Career and Leadership of the NIAID

**DOI:** 10.20411/pai.v9i2.754

**Published:** 2024-09-16

**Authors:** Michael M. Lederman, Neil S. Greenspan

**Affiliations:** 1 Case Western Reserve University, Cleveland, OH

*This interview has been edited for clarity

## MICHAEL M. LEDERMAN, MD

Welcome to *Pathogens and Immunity* Expert Exchange. I am Michael Lederman, and with me is Neil Greenspan. We are editors of *Pathogens and Immunity*, a journal developed to serve the needs of researchers. Our guest today is Dr. Anthony Fauci, who was, until recently, the director of the National Institute of Allergy and Infectious Diseases and the director of the Laboratory of Immunoregulation at the National Institutes of Health.

Dr. Fauci grew up in Brooklyn, New York, where he played basketball for his high school and both stickball and punch ball with his friends in the streets. He graduated from Holy Cross in Worcester, MA, and received his medical degree from Cornell University in New York City, where he served as intern, resident and chief medical resident. He completed fellowship training at the National Institutes of Health, and in 1984 became director of the NIAID. For decades, Dr. Fauci guided our national response to the HIV/AIDS pandemic and, importantly, was the principal architect of the President's Emergency Plan for AIDS Relief, known as PEPFAR, that has saved millions of lives throughout the developing world by assuring access to life-saving antiretroviral therapies.

In recent years, he's been the voice of our national response to SARS, Ebola, and COVID, occasionally finding himself in the difficult position of having to clarify the remarkable statements of our former President. Throughout this time, Dr. Fauci has led a large basic and translational immunology research laboratory, The Laboratory of Immunoregulation, which has revealed a trove of insights about HIV pathogenesis and trained scores of scientists who are now national and international leaders. He has received numerous honors and awards, including the National Medal of Science, and the Mary Woodard Lasker Award for Public Service. It's a real pleasure to have you with us, Dr. Fauci. Welcome to *Pathogens and Immunity*.

## ANTHONY S. FAUCI, MD

Thank you very much. It's great to be with you.

## NEIL S. GREENSPAN, MD, PHD

What early influences steered you to medicine?

## ASF

I think it goes way back to my family background. My father was a pharmacist in a small pharmacy that he owned in the Bensonhurst section of Brooklyn. That was a very long time ago. That was in the 1940s and 1950s. At that time the neighborhood pharmacy was sort of like the hub of the neighborhood advice about medicine. And, unlike the chain pharmacies of CVS and Walgreens of today, it was kind of the doc of the neighborhood: family counselor, neighborhood psychiatrist, and person who you would go to if you didn't have enough money to go to a physician. You could get free advice from the pharmacist. So, the idea about service to others was something that I kind of grew up with from the time I was a child, and then that was fortified by my high school and college educations, which were Jesuit training in a high school in Manhattan — Regis High School and then Holy Cross College in Western Massachusetts. The theme of those educational institutions was service to others, and I felt the best way to serve others in the context of having an affinity for science was to be a physician.

So as soon as I got into high school, I realized that was something I wanted to do. I also wanted to be very close to people, so I took a humanities course, and it wasn't the standard road to a medical school. It was, you know, classics, Greek, Latin, philosophy, together with enough science to get me into medical school. And that's really one of the reasons why I wanted to become a physician, a combination of wanting to be with people, but also having an affinity for science.

## MML

That's great. I wish more of our students had an experience in the classics. You don't find much of that anymore in our schools.

## NSG

I'll just mentioned as an aside that I'm not one of those people who is excessively enthusiastic about so-called STEM initiatives, because I think they're a little bit too narrow, and I think they actually don't work well for a lot of people like yourself who just described how valuable it was to get educated in the humanities along with the science.

## ASF

I think if you exclude one for the other, you miss out on parts of your education. My being steeped in the classics has been very important to me. Being steeped in the classics, I think, helped me after I became a physician to really feel very, very strongly about the problems in society, the problems in civilizations, the problems about disparity — all of that, I think, was helped by my background in the classics.

## MML

So, you went to medical school. You got your degree in medicine. Why did you decide to train in infectious diseases and have a research career in immunology? There were so many choices, why those two?

## ASF

I think it really reflected what my own personal needs are, and things that attracted me. The thing that attracted me about infectious diseases, and I'll talk about immunology in a moment, was wanting to be able to have a target of my work. There are many diseases, that we all are aware of, that have multifaceted components — cardiovascular, immunity, GI, and endocrinology. With infectious disease, you had a target. It was an individual agent that was causing the disease. Usually the disease is preventable, and usually it's entirely treatable; if not entirely treatable, certainly suppressible, as we've done so successfully with HIV.

Also, I wanted a disease in which the stakes are high. I'm not talking in any disparaging way about other subspecialties. I'm talking about what fits my personality. I wanted a challenge in which you have to get it right or the consequences will be dire, and infectious disease is one of those disciplines.

With regard to immunology, when I was in medical school — now, remember, as most people know, I'm a pretty old guy now, so I graduated from medical school in 1966 and started my internship in 1966. At that point, human immunology was in its incipient stages of appreciation, and it was a field that, historically, is closely related to infectious disease. So, I was excited about the knowledge that was rapidly accumulating in human immunology. And since human immunology is so closely connected with infectious disease, one depends on the other, evolutionarily and otherwise, that I decided I wanted to have a combined research and clinical endeavor in infectious diseases and immunology.

And you might know from my history, Michael, I had a combined fellowship in infectious disease and clinical immunology. I'm board certified in internal medicine, in infectious diseases, and in clinical immunology and allergy. And for the first several years of my independence as an investigator, I was more heavily involved in autoimmune inflammatory diseases where I developed therapies for some of the vasculitides. And credit to my mentor, Shelly Wolff, who put me on that project. It was a highly successful project that took diseases that were uniformly fatal, and we were able to induce 90-plus percent remissions. I did that for several years, until HIV came along, and then I completely turned around the direction of my career to study exclusively HIV in its very, very early years. Right from the summer of 1981.

## MML

Your decision to go into immunology and infectious disease (ID) was obviously prescient — clearly prescient. And in those days, and I remember them well, because I'm only a bunch of years younger than you are, the ID docs were the best clinicians around, which is one of the reasons why a lot of us decided to do ID, but they all studied bugs and drugs. There were precious few ID physicians who had made the decision to be immunologists. So good choice.

## ASF

Well, I think it was, retrospectively. It was a good move.

## NSG

Are there any particularly consequential or unusual insights that you believe helped to guide your clinical and research careers.

## ASF

I have to jump ahead a few years from the end of my training, Neil, and it's something I just mentioned a moment ago. When I saw the first MMWR in June of 1981 of 5 young, otherwise healthy, gay men from LA who presented with pneumocystis pneumonia [1], I thought it was a fluke. I was very curious about it, but I thought it was some kind of drug they took that suppressed their immune system.

Then the second MMWR in July of 1981, which now was 26, curiously and phenomenally, all young, otherwise healthy gay men, who are presenting not only with pneumocystis, but with Kaposi's sarcoma, and with other opportunistic infections, who now were not only from LA but from San Francisco and New York [2], that was a particularly transforming moment in my career and in my life. I said to myself, apropos of what we were talking about, the relationship between immunology and infectious disease, here was a brand-new disease. And if you look historically, when was the last time we found a brand-new disease? I mean, like never. It was just one of those things that this was a historical moment.

It's a brand-new disease. We knew at the time, epidemiologically, that it had to be an infection, even though we didn't know what the etiologic agent was. We knew it was destroying the immune system, and it was killing people. So, for all the reasons that brought me into infectious disease, it checked every box in spades. But also, I was trained as an immunologist and in infectious disease. And here is an unknown infectious disease that's destroying the immune system and killing people. So, it was like, Oh, my God! I have to study this disease. And it was really interesting, because when I made the decision to turn around the direction of my career and give my autoimmune vasculitis project to someone else that I had trained in the previous few years, my mentors thought I was making a mistake, and they were wise mentors. They said, “You're foolish. Don't give up your day job. You have an accelerating career where the trajectory of your career is very steep. Why do you want to start doing this, which likely is going to go away and not be of much consequence?”

And then, obviously it became clear very soon, even to my mentors, who were wise enough after a while to say, “I think you were right in making that change.”

But that was a transforming moment in my career. That second MMWR in July of 1981.

## MML

I got similar advice from some of my seniors when I decided to get involved in HIV research in late 1982, a bit later. But they said, “This is going to go away. This is not going to be an important thing.”

## ASF

It just goes to show you.

## MML

You bet!

## NSG

I had intended to ask what specific insights you gained in terms of your success with developing the treatments for the vasculitides? As I recall, when I first heard about you, it was in the context of Wegener's granulomatosis.

## ASF

Yeah, which is now called granulomatosis with polyangiitis. That was an interesting point in my career. I give a lot of credit to my mentor, Shelly Wolff. And the younger people in ID probably don't know or remember who he was, but he was really one of the real giants in infectious diseases, along with people like Bob Petersdorf, people like that.

Shelly put me on a project where we made a decision to do something that was very bold. We had experience as infectious disease docs. So even though I was doing an autoinflammatory autoimmune study, I still was the infectious disease consultant along with my other colleagues at NIAID for the other NIH Institutes. And we would get called often to the National Cancer Institute, whose physicians were giving combined chemotherapy for lymphomas, and Hodgkin's, and lymphoblastic leukemias, and one of their combined therapies always included high-dose cyclophosphamide. So, we thought, wouldn't it be interesting if we could use low-dose daily oral cyclophosphamide at a dose low enough to suppress the aberrant immunological response, but not enough to cause such leukopenia that you put them in danger of infection?

So, we monitored their white counts and kept them within a safe range but continued on a daily basis. And to our amazement, the remission rate went from 0% to 93% with these vasculitides.

And it showed for the first time that you can use a drug that's used for cancer at lower doses for non-neoplastic diseases. It taught me that sometimes you have to take majestic leaps and take chances and the risk is high, but there's a (potentially) really high benefit.

## MML

When I think back to the early days of the AIDS epidemic and how horrendous it was, and how all of our patients were dying in front of us, and our interventions might slow things down just a little bit but not enough. And all of our patients, most of our patients were dying, and there was an outcry of outrage by activists all over the country who said we weren't doing enough, and we weren't doing it right. Somehow you managed to establish a collaboration between AIDS activists and researchers, which was unusual but very successful. How did you do it? And what were the challenges in doing it?

## ASF

Let me try to give some succinct background for our listeners, because I think it's important. Early on, in the early to mid-eighties, we had a scientific community and a regulatory community. The scientific community is the NIH, and people like you and I. And the regulatory community was the FDA. And we were doing things for decades, even before you and I were players in this game, and it was a very rigid approach towards clinical trials. There are inclusion criteria and exclusion criteria. There's no possibility of getting access to a drug outside of a clinical trial, and when it got to the FDA, it would take them several years to approve an intervention. Well, this was for diseases that it worked very well for, but HIV was different. Because it was a different disease, this approach was ill-suited, and the activists knew it. And they were saying, we don't think you're doing enough. But, more importantly, we want to be part of the discussion. We want a seat at the table so we can talk about the design of clinical trials. We want to talk about the rigidity of the regulatory process, and we want to talk about the fact that we need to put in more resources. Well, the scientific community at the time said, understandably but completely inappropriately now that you think about it, “We know what's best for you. We are the scientists, we are the regulators.” And then when I say that now, it gives me chills because of how inappropriate that was. So that's when they said, “Okay, we're going to start being confrontative, disruptive, and iconoclastic until you start listening to us.” And that's what they did. And since I was a public figure, and was one of the few people back then, who was very visible with HIV/AIDS, I became the target of their confrontative behavior. And when they started to attack me, I said to myself, let me listen to what they're saying, because these guys are in great pain. They're angry and they're afraid.

So, I found out that when I put aside the theatrics, and I put aside the iconoclastic behavior and listened to what they were saying, what they were saying was making perfect sense. And I said to myself, if I were in their shoes, I would be doing exactly what they were doing.

And that's when I said, let's sit down and listen to you. And once I did that, I invited them in to sit down, which surprised them — that's the first time any government official ever said, let's sit down and talk about it. And that began a relationship, which didn't immediately become a love affair. It took weeks and months and years to gain their confidence and now, fast forward 30-plus years, the activist community and the advocacy community is an integral part of everything we do about the advisory committees, about the community programs. So, we now listen and we're better off for it, but it took a leap to say, even though you're attacking me, I'm going to listen to you. And that, I think, was one of the best things that I've ever done was to make that decision.

## MML

I have to say, in the trenches, it was incredible to all of a sudden find ourselves in these committees, in these groups, in the AIDS Clinical Trials Group, working directly with folks in the community who were committed, knowledgeable, thoughtful, and the collaboration was, I think, highly successful. At a great level, it contributed to the enormous success of combination antiretroviral therapies that now have rendered survival close to that of the general population. And so, we've got other challenges now. And the big challenge that the activist community and scientists are worrying about is the cure. Are you optimistic that there'll be a scalable and safe cure strategy at some point in the near future?

## ASF

I'm by nature a cautious optimist. If I wasn't, we wouldn't have taken the chances we did over the many years of my career. I think it's a high bar and a tough situation.

I mean the idea that we keep hearing yet again another cure by a stem cell transplant. With all due respect, I don't need another one to tell me anything. We've had enough. We know that you can do it. But the idea of doing an ablation and a stem cell transplant is not the way to go. So, the idea is, can we figure out a way to eradicate the virus? And if not, can we keep it under wraps without necessarily giving daily antiretroviral therapy? To me, the closest thing thus far, which isn't a cure, is giving an injectable every several months. Now that's not a cure in the eradication sense.

But, boy, it's pretty close, when you can give a medication just every few months and keep a virus very, very, very well suppressed. That doesn't mean at all that we should give up on trying to figure out a way to either excise or silence any other reservoir viruses without giving an antiretroviral. I think we should pursue that and continue. If we're successful, wonderful. If we're not, there are other means to keep people suppressed with relatively little toxicity.

## NSG

Is your reason for saying that stem cell transplant isn't the answer one of cost and scalability? Or is it on some other basis?

## ASF

Cost and scalability is important. But if I am a person living with HIV, and I'm either taking one pill a day with little toxicity, or I opt to go for an injectable every several months. Why would I want to take the chance of getting an ablation and a stem cell transplant where you have a mortality rate that is way greater than my one pill a day. Why would I want to do that?

## NSG

Is the injectable actually available now?

## ASF

Yes.

## MML

And, it also can be used for protection/prevention.

## ASF

One of the things that everybody is excited about, myself included, is lenacapavir. The study that was given in cisgender women — 100% protection, wow [3]! You don't see that very often.

## NSG

The question I have relating to vaccines is, what are your thoughts about the prospects for developing a truly effective vaccine for prevention in the next 5 or 10 years. And do you think there's any hope in terms of the antibody approach versus the CTL-based approach?

## ASG

Put it this way. When we did the first beginnings of a vaccine trial back in 1987, 1988, when we had naively given an envelope protein that we thought was going to do what most other pathogens do — elicit a response with a neutralizing antibody to an envelope — it didn't happen. Fast forward a few decades of multiple failures, as it were — we don't like to use the word failures, because you always learn something from a trial — but trials that did not give us the result that we needed.

If you look at some of the research that is now coming out of laboratories, like Bart Haynes and Dennis Burton and Ian Wilson — people like that, where they're really right now able to engage the germline B cells and with multiple sequential immunizations, you can mimic the evolution of broadly neutralizing antibodies.

It's really, really tough. And the latest stuff that has come out from those labs isn't there yet. But, boy, it's going in the right direction. So, although I think it's going to be difficult, I think the key word you mentioned Neil was in the next 5 or 10 years. It's not going to be next year. It's more likely going to be 10 years than 5 years, but I think we have the best minds in a combination of immunology and retrovirology working on it right now. And I am cautiously optimistic that we'll get there.

## NSG

I followed that work a fair amount. And the question I have, and I actually published a commentary some years ago about this [4] is can you really imagine scaling up a vaccine based on multiple different immunizations on the scale of millions to tens of millions of people? Maybe it could be more focused. But I think it's going to be a major challenge, especially given the social context.

## ASF

You're absolutely right. I think it is a major challenge. But is it technically, scientifically possible? I think so. I've always felt that once you conceptually prove something, then it becomes an engineering problem of how do you get it to be scalable? So, if we could conceptually prove you can do it, it's kind of like taking the first big computer that fits in a big room and then turning it into something I have here on my cell phone. That's what I'm talking about. Let's conceptually prove it first.

## NSG

Well, and just as an aside, an interesting example of this sort of problem is you, no doubt, were among those who saw the article by Francis Collins about his sort of devastation over the lack of utilization of the medications that cure hepatitis C. And we actually interviewed as one of the people in this series, Ray Schinazi, who had a major role in developing those drugs. And there's really an example of what you said before about the potential to actually effectively treat, in this case amazingly cure, a viral infection — a huge percentage, ~95% of patients who got the drugs, and yet it's being very much underutilized, at least as Francis Collins described it in his op-ed in the New York Times [5].

## ASF

He was absolutely correct. It is tragic. As you said — oh, my goodness, we're actually curing an infectious disease that causes cancer that is incurable once the cancer occurs, like holy smokes. Wow.

## MML

The AIDS treatment strategies are fantastic. Most people recover their CD4 T cells and their functional immunity, but a minority remain compromised. It happens sometimes. Do you think there's a way to enhance immune competence in those people? We tried IL-2. It increased CD4 T cells, but they didn't do what we wanted them to do. Do you think that additional studies of other cytokines, like IL-7, are warranted, or other strategies to bolster immune responses?

## ASF

The answer is, yes. But again, I say that cautiously, because what we've learned from the IL-2 studies is that when HIV comes along and punches out at the clonal level, antigen-specific clones, you could expand all you want, but if the clone isn't there, you're not expanding the right clone. So I think you can get some degree of reconstitution, but I'm not so sure how much. But that would not prevent me from still trying other approaches.

## NSG

Moving to the genetics of HIV resistance, have you given any thought to why it is that the CCR5-Delta32 mutation is as prevalent as it is in large parts of Europe, and what is your assessment of the various studies that have tried to indicate that there's a selective advantage for that allele in perhaps some other infection?

## ASF

That's not my field. I've just followed it, and there has been, as you well know, proposals that somehow or other a disease centuries ago — smallpox or some related disease — in which the selection for those who had that mutation essentially kept those people alive by protecting them against the thing that was killing them and selected to have a population — for example, Northern European Caucasians much more so over Southern African Blacks. So there had to be something in history where that allele was protective against something that was killing large swaths of the population. But, like many others, I don't know what that disease is.

## MML

So COVID shows up, becomes a pandemic, and amazingly an effective vaccine was developed incredibly rapidly. What are the implications of that for us?

## ASF

There are a lot of major implications and collateral implications. The major implication is the importance of investment in basic and clinical biomedical research. Because if you look at the investments that were made in the mRNA platform technology, you go back and look at the first paper that modified the mRNA to allow it to avoid an inflammatory neutralization, to be able to be used as a vaccine was a 2005 paper in *Immunity* by Katalin Karikó and Drew Weissman [6]. That was like 15 years ahead of time. And then, if you look at Barney Graham's immunogen design of the S2P mutation that stabilized the spike protein in its prefusion form that made it highly immunogenic [7], they were working on that for decades for HIV. Barney was working with Peter Kwong, who was trying to get the right cryo-EM conformation of the envelope and Barney used that cryo-EM approach to get the right stable conformation of the spike protein, which he started off with MERS, and then, as soon as COVID came along, he immediately took SARS-CoV-2, and it took him like a few days to be able to show that and put a mutation in [8]? So, it tells us about the power of basic and clinical biomedical research.

But the other thing it tells us is something very elementary. When the body shows you that it can make an adequate immune response that can clear the virus and protect you, then that is a road-map to a vaccine.

If you get infected with measles or infected with polio, even though there's a degree of morbidity and mortality, the overwhelming majority of people survive and are left with immunity that protects them against reinfection. And when you have a pathogen that doesn't change much, like measles and polio, you can use the body's natural immune response as a roadmap to developing a vaccine. That's exactly what happened with COVID.

The body induces a very good immune response against SARS-CoV-2, which is the reason why the overwhelming majority of people recover from COVID, and the SARS-CoV-2, they eliminate it from the body. And this gets us back to the question you asked a moment ago about HIV. The reason why it's so tough to make a vaccine against HIV is because the body's natural immune response to HIV is not adequate. There are no examples of spontaneous clearing of the virus. There are a lot of elite controllers who control it, but they don't clear the virus. So, if you take that conceptual proof that it could be done. And then you take Operation Warp Speed, where you say the federal government will make an investment of billions of dollars to de-risk the companies to go in and make a major investment in doing the clinical trials in collaboration with the NIH, with the clinical trial groups, the HIV Vaccine Trials Networks, and those others to collaborate with them, but to also guarantee the company will not lose any money.

So, what Operation Warp Speed did is not only did it finance and bankroll clinical trials of 30,000 people per trial, but it pre-manufactured the vaccine before we knew it even worked, so that as soon as the clinical trial showed that the vaccine was highly effective. Bingo. We have tens of millions of doses of vaccine available to go. So, it's got to be investment in science and bold investment in combination with the pharmaceutical companies.

And the reason I say that is we can do that with any disease.

## MML

Citizens need to recognize that, and that being prepared is really key. And this was an enormous success, and I guess the classic term would be *semper paratus.*

## NSG

Are you aware of any pathogens or circumstances clinically that might make mRNA vaccines less advantageous than maybe older technologies?

## ASF

No, the only thing that is curious is that I think we need to see how it does with other pathogens — because right now obviously, there's a lot of work trying to apply mRNA to flu and apply mRNA even to HIV. One of the things that is curious is the duration of the protection of a mRNA vaccine. Now, I don't know whether that's a peculiarity of a coronavirus or the peculiarity of the mRNA platform. But we know when we vaccinate somebody, the protection is not measured in decades. It's measured in months to a year. That's the issue that we've got to work out.

## MML

So, with regard to that issue and something you mentioned earlier, how should we define herd immunity with respect to COVID, I mean, how do we think of it?

## ASF

I wrote a paper on that. It was a simple paper [9]. It stated that we cannot apply the standard criteria of herd immunity. It's not applicable with SARS-CoV-2. And the reason is, it's simple. I can synopsize the paper in 30 seconds. One is that herd immunity is dependent on an immune response that is durable, measured in decades to a lifetime, and a pathogen that does not change. So, you have clear-cut herd immunity with measles. Why? The measles that I got infected with as a child, because I was born before the measles vaccine, is the same measles that's killing kids in the developing world today.

Number 2, if you get infected with measles or you get vaccinated with measles, the duration of protection minimally is decades and maximally is lifetime. Those are the criteria that you need for herd immunity. Because if you have a pathogen that keeps changing like the multiple variants of SARS, and if you have a duration of immunity that's measured in months, the entire concept of herd immunity is no longer valid. That's the point.

## NSG

This gets into the next question, which is — and I know you've been asked this by other interviewers, but I think it's important to address how precisely to think about the effectiveness of the mRNA vaccines in limiting transmission, because people often talk about it in absolute terms that it either does or doesn't, and I want to know if you would agree that — my take would be that even if it isn't 100% able to prevent transmission, that doesn't mean it's not benefiting us by reducing the probability of transmission, or the scale of transmission.

## ASF

No, I'm with you on that, because it doesn't do it to the 90% or even 80% or 70%. It does have some impact on transmission, number one. However, that impact is short lived. So, if you start off with the ancestral strain that we made the vaccine against. In that trial, it likely had a reasonably good effect on transmission, not 93%, but reasonably good.

What we learned from experience, and you know the people who criticize the scientists and criticize the public health officials say, “You told us it was going to protect.”

We made an assumption, that protection would remain at a high level, and it didn't. It was lower than we wanted to begin with, and it didn't stay very long. What stayed long was the protection against severe disease leading to hospitalizations and death. That was reasonably durable, not measured in many years, but durable beyond a few months; whereas the protection against infection was lower than that against severe disease and was much less durable. But in answer to your question, that doesn't mean there was no protection against infection. If you could get a little bit of mileage out of that, it would be worth it. So long as you don't — now that we know what the results are — you don't say it definitely is going to protect you against infection. It's not. I'm a classic example of that. I've been vaccinated 6 times with the primary series, followed by a bunch of boosts. I've been infected 3 times.

So not only does vaccine (not fully protect), but hybrid immunity with vaccine plus infection didn't protect me against getting infected. Three and a half weeks ago I had a very mild infection, but I still got infected. Now, given my age, if I didn't have hybrid immunity, I might have died from the infection.

## MML

We're trying to prevent this with immunization and social efforts. What about masks? How effective are masks —if we wear them, if we wear them well or if we wear them not well —how good are they?

## ASF

Thank you for asking that question, because there's a lot of misinformation and disinformation. A properly fitted mask worn consistently — of an N95, and to a lesser extent a KN95 — clearly is protective [10].

There are studies now that clearly take the fog away from the question: Do they protect or not? They clearly are protective. Are they100%? No, of course not. Because people wear masks, and then every once in a while they take them off because they have to go eat on a plane. People say, “Well, I wore it for the entire time, except when I was eating.” Well, sorry about that.

## NSG

What about the case of mask mandates, are they worth trying? Clearly, it must be the case based on what you just said, and that was my own expectation: that they're effective if worn properly. When we're applying it on a mass scale, at least in the social context in the US, is that going to work in the future?

## ASF

Well, I think the mood of our society has said, in a perfect world, if you had a society that didn't have that strain of independence — you can't tell me what I'm going to do with my life — that mask mandates would work just like other mandates, because it would get virtually everybody to wear a mask. I think the pushback against any mandate of anything thus far may turn out to be counterproductive, unfortunately.

## MML

Heartbreaking, isn't it? So, we had this pandemic in 2020, and we might have another one in time. Are we prepared? Do we have a good system for sentinel surveillance in our country that will pick up something quickly and tell us who it is and what we might be able to do about it?

## ASF

Well, I'm sorry to say that I don't think we're as prepared as we could be given the painful experience we've been through. I look upon pandemic preparedness and response in two buckets. One is a scientific bucket, and the other is the public health bucket, and I think if we continue to build on the high A-plus success of our scientific preparedness and response — as proven by getting a highly effective vaccine in a record time beyond anyone's imagination of 11 months from the time the sequence was made public on a public database, by rapidly developing monoclonal antibodies; if we can do all those things that were done — if we continue to do that, we're good.

What we really need to do is to improve our public health response, because the fractionation (that now exists) between individual patient care, going into an ER, going into a clinic and the public health that is sort of guided by central organizations like the CDC.

You know, the local groups are not obliged to report things to the CDC. The CDC doesn't have power to say, “You've got to give us all this information in real time.” We don't have complete electronic medical records the way they do in some countries where in real time you could know exactly what's going on in the community. We've got to do much better in that. We've got to build up the local public health system so that we have good coordination between the central and the local, and we can respond in real time. We didn't do a very good job with that.

The other thing that is a lesson learned and I don't know how we're going to change this, because it's become sort of like a landmark of our society. But the worst time you could ever have a pandemic is when you have a profound degree of divisiveness in society, where people make public health decisions based on political ideology. The idea that when Trump said, “The CDC is recommending masks; for me personally, I'm not gonna wear one.”

So, what that did was it immediately made masking a political issue, so that if you are supporting the president, you don't wear a mask, and if you're not supporting the president, you wear a mask. So, there were fights of people wearing masks or not wearing masks.

That's a public health decision. Why should people be fighting about that? Because it had a political tinge to it. So, as long as we have that, we have trouble. Let's look at the deaths and the hospitalizations: the red states and Republicans versus the blue states and Democrats. The deaths and hospitalizations associated with COVID were very heavily weighted towards red states because they under-vaccinated versus blue states because they vaccinated more [11]. That should never be. There should never be avoidable deaths because of decisions made because of political ideology. That is so tragic.

## NSG

I don't know if you saw this, but two years ago there was an article in *New England Journal of Medicine* about this initiative to have a safe and effective vaccine within 100 days for any new putative pandemic pathogen [12].

And, I have some reasons for wondering whether this is really plausible. And I think you provided some basis for understanding why extrapolating from SARS-CoV-2 and the experience with the mRNA vaccines is not necessarily fair for all conceivable pathogens, because, as you alluded to, work that had been done on HIV and, even more closely, SARS-1 in the earlier 2000s, provided an exact roadmap for what mutation to make, for instance, in the spike protein to stabilize it.

And so, this group had this paper in *New England Journal*, and I think they were operating on the assumption that you could just sequence a virus and know which gene was encoding a key antigen and stick it in a vector with mRNA and have a vaccine that's safe and effective. And I'm curious to know what you think of that.

## ASF

Yeah. Well, the answer is, that's wishful thinking in some respects. But what I think they mean is that if you look at the prototype pathogen approach that Barney Graham and colleagues wrote about a couple of years ago [13], it's to take the 7 or 8 most likely viral families — alphavirus, bunyavirus, coronavirus, filovirus, all the flaviviruses, etc. — and to make a prototype vaccination and immunogen with that, put it in a phase 1 trial to show that it induces an appropriate immune response, and then, if you get a virus from that family to make the rapid adjustments immediately, and then use the phase 1study that you've already done to jump right into an efficacy study.

The only thing about the 100 days that is a little iffy — I think you could probably identify what you want to do, get it in a vector, and start a clinical trial, and in 100 days have a vaccine that you probably, if you have enough cases, can show is effective, at least in a phase 2 or phase 2B trial. I don't know whether you could do a 30,000-person phase 3 trial, but you may get some information from a phase 2 or phase 2B.

The thing that is going to be tough is to have enough vaccine in 100 days to give to everybody, because that's a production issue. It took 300 days to do that with Operation Warp Speed. I'm not sure you're going to be able to do that in 100 days, but you could probably have in your hand a vaccine that you're pretty sure works, and then you could start distributing the doses as it comes from the manufacturers.

## NSG

My concern would be that it's basically an example of inductive reasoning, and in your experience at Holy Cross, you may have come across David Hume. He pointed out the limitations of inductive inference and I don't doubt it could work. But I don't think we can be confident 100% of the time that we might not be surprised by some virus that's a member of a known family, but in some way has diverged in important respects.

## ASF

Right. The thing you're betting on Neil is that if you take the prototype from that family and you make an immunogen, then that immunogen would be similar enough in its conformation, that if you pick the most immunogenic form of that immunogen, it would apply to whatever the particular outbreak immunogen is.

## NSG

Right. And also, that it doesn't have unfortunate cross reactions.

My linked question comes out of a podcast I heard with this very young but very impressive investigator at MIT named Kevin Esvelt. He was saying that because of the risks of doing experimental work on putative or likely pandemic-potential viruses, in particular, that we shouldn't do any experimental work on those kinds of pathogens.

And it's sort of along the same lines, as I just argued. I'm very skeptical that you can be confident you know what you're doing with a new virus, even if it's part of a known family, with no experimental work in animals. And I'm curious, you know.

## ASF

I agree. I have to respectfully disagree with that that approach, but people get swept away by gain of function, and they usually have no idea what they're talking about: what the definition of gain of function is.

## NSG

That's actually not easy to define.

## ASF

Well, you know, they tried to do that when they were trying to make it less confusing. Hopefully, they'll straighten it out a bit more now, but the operational definition some time ago was well accepted. They want to re-look at it now, that's fine. But the issue about not doing any experiments in an arena to look at predicting what might evolve into an outbreak is foolhardy. That doesn't mean that you can willy-nilly go in and start having pathogens manipulated by people who don't know what they're doing, who are not trained, in which the result doesn't give you any information that's helpful. But you've got to get a group of people with outside influence from the community, scientists of good faith, to say what is the risk-benefit ratio of doing an experiment? And if the feeling is that the risk is too great, don't do it. But don't completely ban studying anything that would have to do with an emerging infection. That would be wrong.

## NSG

What he was talking about was any experimental work, not just putative gain of function research. But about the pathogenesis of a particular virus, which again, you know, even a tiny mutation can shift how a virus deals with the host. And you see this with SARS-CoV-2. Even within a strain, the outcomes in different people, and the nature of the relationship between virus and host is quite variable. It's not as straightforward as oh, we know that family, so we can predict everything.

## ASF

The short answer to your question is that we shouldn't just empirically shut off research of emerging infections. Forget the term “gain of function.” That confuses people. But to say, “let's shut off all research on emerging infection” is really not a good idea.

## MML

A growing part of our community — and some of this is related to political orientation, but not all of it — is reluctant to get immunized. They have a fear of immunization, fear of the side effects, and skepticism about the value of immunization. How should we — physicians, scientists — address vaccine hesitancy in our country and all over the world, in fact?

## ASF

Well, it's a very difficult problem. And one of the things that we've learned is that vaccine hesitancy and not wanting to get vaccinated comprises a broad spectrum of people. From those who are inherently anti-vax, anti-science and those who are hesitant because they need more information. So, you're not going to change the mind of a completely recalcitrant person, who, no matter what you say, is against it. And that person is probably more anti-science than anti-vax.

But there is a substantial proportion of people who are hesitant, that [we can perhaps persuade] if we in a good faith and transparent way reach out to them and try to get them to understand the data and the science associated, and don't sugar-coat anything. If we talk in real terms and say that no intervention is 100% safe, but the risk-benefit of vaccinations over decades and decades and decades has proved overwhelmingly in favor of the positive aspect of vaccines. We just need to keep talking about that and not condemning people who are hesitant. We should not tire of reaching out to them and trying to explain to them in pure, simple, scientific terms and risk-benefit terms why it's important to get vaccinated.

## NSG

One of the issues that I took note of, particularly during the early months of the COVID pandemic, was use of the word “airborne” in describing pathogens. As you no doubt remember, initially, people were saying that SARS-CoV-2 was spread through droplets. And then, I think, over time, eventually it was accepted that it can also spread as aerosols. And there were some people who were not using the word airborne, or didn't think the word airborne applied to droplets, which I find odd because droplets are suspended in air. Do you have any thoughts about whether that term should be used for both situations.

## ASF

I think that if you look at the aerodynamic experts who are now getting involved. They're saying that droplets themselves hang around much longer than we thought they would. True aerosol obviously floats around for very extended periods of time. So, I think that the use of the word airborne is probably going to avoid confusion. Anything that you get through the air, be it a droplet or a classic aerosol, is an airborne pathogen. I would think that sooner or later, if we incorporate that, it's going to be less confusing. The aerodynamics people absolutely insist that this idea that if you have a droplet, it drops in 3 feet, is not so.

## MML

I'm going to switch gears a little bit towards the reason why our journal, *Pathogens and Immunity*, exists. You know we were very concerned, and many people are concerned, about the skyrocketing profits of the for-profit journals on which scientific progress is dependent. There was a nice op-ed in the Guardian a couple of weeks ago by Abizadeh, suggesting that universities start taking on the responsibility for sponsoring not-for-profit journals [14]. Do you think that there's a role for universities to do it. Will Georgetown be doing something like this? Case Western Reserve is right now. That's *Pathogens and Immunity*. Is there a role for universities doing this?

## ASF

I can't speak for Georgetown, because that's not the area that I'm involved with at Georgetown. That would be for other people. But I believe that we really do need to get much more accessibility of journal information to people without the skyrocketing prices. You know, and I know the publishing houses get very disturbed when you do that for sure. But if a young, aspiring researcher doesn't have access to information because they don't have enough money, that's crazy. So, we've really got to figure something out. If the universities are the answer, then let's go ahead and do it.

## NSG

Well, Michael carefully formulated that question to get you to use terminology referring to young people. What I was going to ask next is what advice would you offer to a young person considering a career in biomedical research?

## ASF

The fact that a young person is considering a career means they have an interest in it. What I can tell that young person, as someone who has been involved in biomedical research and now in my 56th year — I was at the NIH for 54 years — that the gratification and the feeling of accomplishment that you can get despite all the roadblocks and the stresses and the strains of getting funded, and some of the anti-science, that it overwhelmingly slants towards the positive. So, if you're considering it, I think it's something you should pursue, because it's amazing how much fun it can be and what a great feeling of gratification you can have from working in the arena of biomedical research.

## MML

On a related topic, physicians sometimes make a decision to also have a biomedical research career, as you did. And it's great to have physicians involved in research, because they see what's happening to patients. And folks who train in basic science, who get a PhD, don't have that opportunity; they don't get that perspective. So, keeping physicians engaged in biomedical research, I think, is a real important priority. But do they need to go to get formal training in science. Does a physician who's thinking about a biomedical research career need to get a PhD as well.

## ASF

The answer, I think Michael, is no, but with some explanation. If you're a physician and you've done your training — a couple of years of internship and residency — and you want to pursue a career in biomedical research and you want to capitalize on your insight that you have as a physician, but you want to be sophisticated enough in the science. You can learn, in a fellowship, fundamental basic science if you get into a lab that is funded enough to give you the time to put into fundamental basic research and learning it. Because you don't want a physician only to be the person that's conducting the clinical trial. You want them to be able to design from a scientific standpoint the important question that needs to be asked from a basic and then ultimately a clinical science standpoint.

I was very fortunate. I don't have a PhD. When I took a 3-year fellowship at the NIH, I went to a very basic scientific laboratory, and I knew that, and I got anxious. I thought, I'm going to lose my clinical creds here. But I didn't. I ultimately got back to being very active clinically. But for those 3 years, I was in a very basic scientific laboratory, and I published in the JEM and JI and those journals. So, I learned basic research. It isn't as intense as a 4-year-or-so PhD program, but it got me enough sophistication in basic research to at least understand it enough to be able to design a fundamentally sound, basic and clinical research projects.

## MML

The path that you took to your career obviously worked very well for you, and very well for us. Thank you so much for sharing your insights and perspective on science, on the epidemics of infectious disease and immunology.

## ASF

Thank you, Michael. Thank you, Neil. It's great being with both of you.

## Figures and Tables

**Figure:**
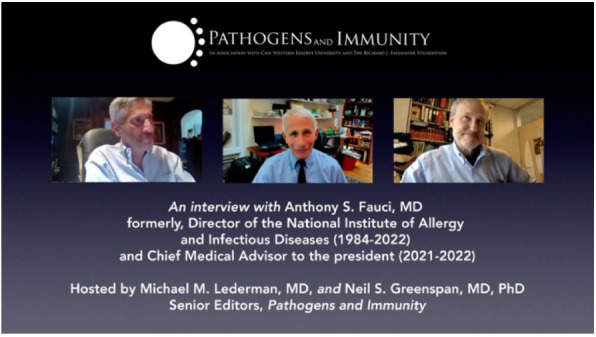


## SUPPLEMENTARY DATA

Supplementary materials are available at the *Pathogens and Immunity* website. Supplementary data may be provided by the authors to benefit the reader. Supplementary data are not copyedited and are the sole responsibility of the authors. Questions or comments related to supplementary materials should be addressed to the corresponding author.
